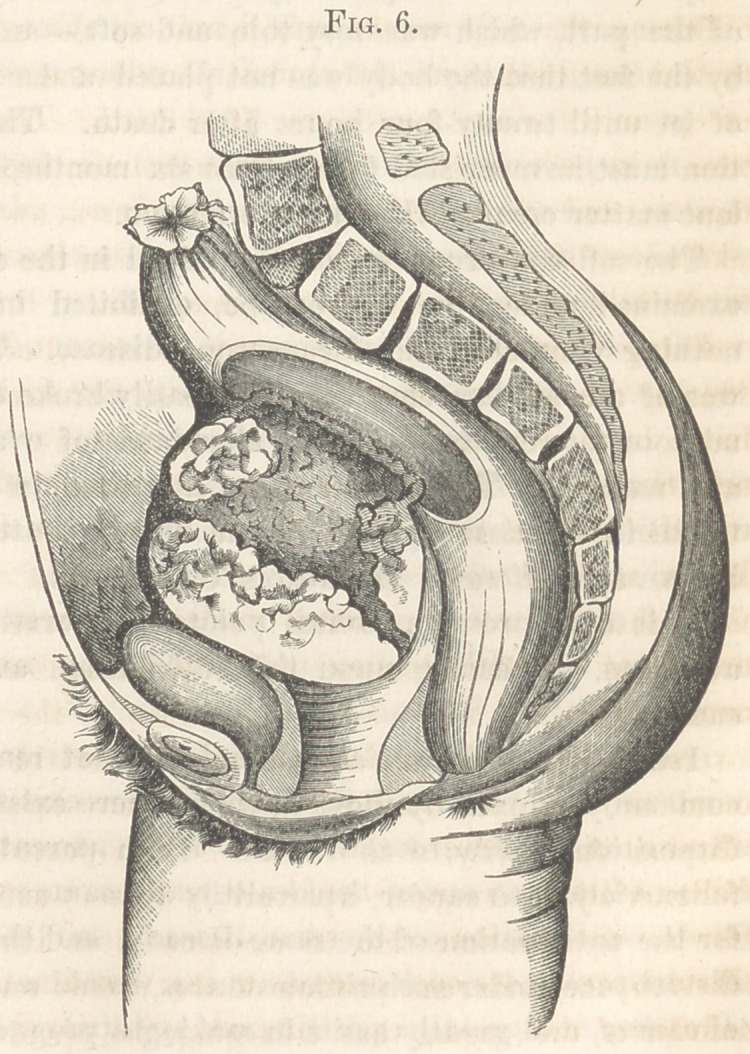# Proceedings of the Pathological Society of Philadelphia

**Published:** 1858-09

**Authors:** 


### ART. III.—*Proceedings of the Pathological Society of Philadelphia.* Reported by the SECRETARY.

WEDNESDAY EVENING, June 9th, 1858.

The President, DR. GROSS, in the chair.

*Dilatation of Heart, with Adherence of the Pericardium; Deposits of Lymph in the Liver; Solidification of the Lung; Disease of the Kidney.*—DR. DARRACH presented a diseased liver, heart, and lung; they were removed from Mr. B., aged nineteen years, who had, when about six years of age, a very severe attack of scarlet fever, which left him much prostrated. His attending physician stated that anasarca had been present. The urine was not examined; most likely his kidneys, at the time, were affected. The patient, however, recovered; but it was not a great while afterwards before he exhibited evident signs of disease of the heart. Since, he has been feeble, and, up to the occurrence of death, has suffered more or less severely from a cardiac affection, yet having sometimes rather prolonged intervals of comparative health.

During his last attack he suffered very much; his lower limbs became exceedingly swollen, so that the skin on his feet burst in several places. There was extended and marked dullness over the heart. No abnormal sounds could be distinguished. The impulse of the heart was diffused and fluttering, but without force of beat. The sounds were not marked nor distant, from which signs the conclusion was, that there existed dilatation of the heart without valvular disease. Posteriorly, mucous and sub-crepitant rales could be heard in the lungs. There was also great dullness on the right side, but no blowing sound could be perceived. The general appearance of the patient was that of one laboring under disease of heart, or albuminuria. The urine was examined a short time before death, and a large amount of albumen was found in it.

Sectio cadaveris.—The cranium was not opened. The lungs were found adherent, throughout their whole extent of surface, to the walls of the chest. The pericardium was in the same condition. Posteriorly, the lower half of both lungs were solidified. The heart was found dilated; its walls not thicker than normal. The pericardial sac was entirely obliterated by the adherence of its approximate surfaces. The mitral valves, and semilunar valves of the aorta were very slightly thickened, but not by any means insufficient. There were extensive washed clots in all the cavities, and at the commencement of the larger vessels.

Abdominal region.—The liver was enlarged, and very firm and elastic to the touch. A cut surface presented brownish yellow and black markings, somewhat resembling a nutmeg liver. The kidneys were larger than normal; the left one was evidently in an early stage of degeneration, being of a yellow hue, which contrasted strongly with the healthy appearance of the other renal organ.

At first sight one was struck with the oily appearance of the yellow portions of the liver; but the color was more of a brownish hue than is occasioned by an oily deposit in that organ; and when, by a microscopical examination, no cells containing oil, nor any free oil globules were found, but, on the contrary, cells less than usually granular, and also much free granular matter, the conclusion that suggested itself, both from the above as well as from the appearance of the organ to the naked eye and its firm, elastic feel, was, that it was not really in a state of fibroid degeneration, but that unorganized lymph had been deposited in the organ, about the capsule of Glisson—perhaps, if the person had lived, to be formed into fibroid cells, but as yet none had made their appearance, or at least could be discovered.

The deposit which produced the solidification of the lungs, no doubt was of the same nature; and the same condition was, likely, commencing in the kidney; a universal lymph deposit having taken place from some fault in nutrition.

*Ulcers on the Tongue, Larynx, and in the Bronchial Tubes; Tubercle in the Lungs and Kidney.*—DR. MORTON presented, for Dr. Hutchinson, a tongue and larynx covered with numerous ulcerations, also tubercular lungs and kidneys. These specimens were removed from C. N., aged twenty-three, a native of Germany, who was admitted into the Pennsylvania Hospital on June 3d, for syphilis, and who died suddenly from œdema of the glottis, on Monday morning, June 7th. When admitted, he was in a state of great debility; his eyes were the seat of a severe inflammation, and he had a very haggard expression; he suffered from diarrhœa; his face was covered with an eruption resembling rupia, which was also found upon other parts of his body; his tongue and palate were horribly ulcerated. It was afterwards ascertained from his physician, Dr. Demmé, that during the winter of 1857 he had contracted a chancre, which soon became indurated. On the 14th of April, of this year, Dr. Demmé describes him as presenting an extremely emaciated appearance, and complaining of soreness in his eyes, a frontal headache, of an uneasiness in the throat, and of a profuse and fetid muco-purulent and bloody discharge from the nose. An examination showed in one nostril, upon the septum, a deep, excavated ulcer, with well-defined edges, at the bottom of which the vomer was exposed, denuded of its periosteum. The course at that time adopted was a mercurial treatment, and injections into the nose of a solution of chlorate of potassa; subsequently he was placed upon the iodide of potassium in five-grain doses. About the 4th of May his eyes became very much inflamed, his appetite failed much, his breath was foul, and he complained of great uneasiness at the throat; but the disease in the nose he declared to be cured. Iron and bark did not remedy the progressing debility, nor did any applications stay, excepting temporarily, the disease in the throat. His eyes, after having greatly amended, became again suddenly much worse, presenting most characteristically the appearance of scrofulous conjunctivitis and corneitis. The lungs, which were at that time examined, sounded clear on percussion. There was no cough, nor were there any symptoms which pointed to the liver or kidney. Toward the middle of the month, rupia-like ulcers appeared upon the skin, and on the mucous membrane of the mouth and tongue, and an exhausting diarrhœa set in. It was in this state that he was carried to the Pennsylvania Hospital.

Post-mortem examination eight hours after death.—The tongue was one mass of distinct ulcers from end to end; the entire top of the organ was eaten away. Some of these ulcers appeared, on close inspection, to be situated on the surface of an exuded whitish matter, or a degenerated substance, and not directly on the tissue of the tongue itself. They extended, forming a continuous line, through the larynx into the smaller bronchial tubes. The tip of the epiglottis was destroyed, and also the vocal chords; the lungs were filled with infiltrated tubercle; tubercular ulcers were situated throughout the small intestine; the kidneys had large patches of infiltrated tubercle scattered throughout their structure. The cortical and the tubular tissues had, in parts, entirely disappeared. In the left testicle was a small softened spot, which had the appearance of a commencing abscess.

DR. MORTON directed the attention of the Society to this case as combining lesions produced by syphilis, with tubercular disease of several organs. It was difficult to say which of the ulcerations were produced by syphilis. The ulcers on the tongue and larynx might be syphilitic or tubercular.

DR. GROSS inquired if the ulcers on the tongue had an indurated base.

DR. MORTON stated that it had seemed to him as if the tissue around the ulcerations was dense and hard.

DR. BRINTON asked if the glands of the neck were indurated.

DR. MORTON had not perceived them to be.

DR. KELLER thought it a difficult matter to determine whether syphilis had preceded or followed the tubercular deposits. From examining the specimens on the table, he was inclined to believe that the tubercular matter had been deposited in the different organs at different times. The disease of the kidney was evidently much older than that of the lungs.

*Perforation of the Aortic Valves.*—DR. MITCHELL presented, for Dr. Darby, a specimen of disease of the aortic valves, with Dr. Darby's account of the case. The patient from whom it was obtained, Thomas Darley, a boatman, thirty-five years of age, was admitted, on Thursday, May, 21st, into the St. Joseph's Hospital, exhibiting signs of great suffering and debility. His face was pale; his lips were colorless; his eyes heavy; the extremities cold and clammy; his forehead was covered with large drops of sweat, and his hair as moist as if he had been under a shower-bath. While making arrangements for entering him as a patient, he complained of so much weakness and nausea that half an ounce of brandy was administered, and he had to be assisted into the ward and undressed by an assistant. On being placed in bed, he immediately vomited about six ounces of fluid, resembling coffee grounds, covered with froth, and of a sour, fetid odor. In the ejection of this fluid he suffered intense pain, and complained of great burning in the region of his stomach. Being for the time unable to answer questions, he gave, after a period of several minutes, a rather disconnected history of his case. He had been quite suddenly taken sick on the Friday previous, with great weakness, much nausea and pain in the epigastric region; he did not think it would last, and went on board the boat in New York, but found, on the first day of the passage, all symptoms increasing, so much so that he could not do duty; and, on going to bed, was unable to lie on his left side. His bowels at first were loose, and a cough soon commenced, which produced great pain over the whole of the hypochondriac and epigastric regions and left side of the thorax; he expectorated thick, white, frothy sputa, without any discoloration. He had been unable to eat anything since he was taken sick, on account of constant vomiting, but he drank a great deal of water, as it cooled the burning in his stomach. From intense pain in coughing or drawing a long breath, he had not slept since the attack; no medicine had been taken; but to relieve pain he had placed a tar plaster over the region of his stomach.

On examination, his pulse was found threadlike, and beating 104 in the minute; his tongue was dry and thickly coated, and his whole abdomen was tympanitic; the stomach was much distended, felt hard, and was exquisitely painful to the touch; there was much dullness, on percussion, on the left side of the chest, particularly over the region filled by the posterior lobe of the left lung, and on the left side the respiratory murmur could only be heard beneath the clavicle. On the right side the respiration was puerile. His heart palpitated violently, and a bellows murmur could be heard over the aortic valves, and as high as the arch of the aorta; his breath was so fetid as to render it unpleasant to remain near him; his cough was harsh, dry, and frequent, causing great pain during the paroxysms, which were of some length.

From this time the patient lingered for two days, suffering from symptoms of gastritis, and annoyed by vomiting and great oppression of breathing. The vomited matters were composed of mucus, bile, and altered blood; his pulse ranged between 90 and 100. Death took place suddenly, about twenty-four hours after his admission, and while he was in the act of turning over.

Post-mortem examination eighteen hours after death.—The heart was much enlarged, and the auricles were relatively more dilated than the ventricles. The only valves found diseased were those of the aorta; the edges of two of the discs of these valves were much thickened, and the valves were pierced by three perforations; the muscular structure of the right auricle was separated in longitudinal sections, as if cut by a knife, and was only held together by connective tissue. There was no actual perforation of the auricle, for no blood was found in the pericardium; yet it was so thin as to present the appearance of being ruptured; and not until the auricle was distended, by water being poured into it, was the fact proved otherwise. There were two quarts of watery effusion in the cavity of the thorax, compressing the left lung to one-third the natural size. In other respects the lungs appeared healthy. The stomach was filled with a dark and fetid fluid, and the structure was much changed, being emphysematous when rubbed between the fingers. The mucous membrane was elevated, decomposed in parts, and much inflamed in the middle portion, the inflammation extending toward the pyloric orifice. This inflamed portion was traversed by enlarged vessels, and in other parts of the stomach dark-greenish colored spots were seen. The liver was decreased in size, and such considerable force was required in tearing it, or pushing the fingers through its structure, as to induce the opinion that there was cirrhosis. The other viscera presented no abnormal appearances.

---

WEDNESDAY EVENING, June 23d, 1858.

The President, DR. GROSS, in the Chair.

*Syphilitic Ulceration of the Larynx.*—DR. HALL exhibited a specimen of syphilitic ulceration of the larynx. It was obtained from an Irish woman, unmarried, aged thirty-six, of robust frame, but of a worn, slightly haggard expression, who was admitted into the medical ward of the Pennsylvania Hospital, August 31st, 1857, for laryngitis, under the care of Dr. Gerhard. The most prominent symptom, and one that directed attention immediately to the site of the disease, was the harsh, stridulous respiration. Her pulse was good, and there was none of that purplish appearance about the face to indicate much obstruction to the circulation in the lungs. Percussion revealed no dullness; over the front and back portions of the chest there were merely some few bronchitic rales to be heard.

Owing to her dyspnœa and apparent reluctance to answer questions, not much information could be obtained from her, beyond the fact that she had been sick for four weeks—the result of a cold she had taken. With a view of promoting diaphoresis and relaxation, vin. ipecac. in syr. scilla was ordered every two hours. She was placed upon a good supporting diet, with beef essence and broth.

At the evening visit, the dyspnœa still continuing, calomel, half a grain, and nitrate of potassa, ten grains, every two hours, were ordered. A blister, two inches square, was applied over the throat.

At midnight she was breathing very much better; there was very little stridor; she was sleeping, and seemed to be much improved.

At 4 A.M., of September 1st, Dr. Hall was aroused in haste, but, on reaching the bedside, found that the patient was dead.

Examination ten hours after death.—Head, nothing abnormal. Lungs and heart perfectly healthy. The liver, stomach, spleen, kidneys, bladder, and intestines, also healthy. The right labium externum was the seat of two excavated chancres with indurated bases.

The immediate cause of death had been œdema of the glottis and of the rima glottidis. In the larynx were two ulcers, each the size of a ten-cent piece; one just below the upper border of the thyroid cartilage in the median line, superficial in character; the other, situated over the cricoid cartilage on the left side, extended deeply, and had produced corrosion of the cartilage, and formed an abscess external to the larynx in size, of two and a half inches by two inches in breadth. The walls of the abscess were much thickened, and contained a small quantity of thin, fetid pus.

This case is one of interest, from the nature of the affection, and from the light thrown upon it by the post-mortem examination. The ulceration of the larynx might be mistaken for tubercular deposit; the absence of tubercle in the lungs and other organs, together with the inflammation and destruction of the cartilages of the larynx, went to disprove this—the presence of the chancres on the labia forming conclusive testimony as to the character of the ulceration in the throat.

Langston Parker calls it one of the most formidable varieties of constitutional syphilis, and says that he has seen several examples of it. Ricord, Cazenave, and Carmichael, of Dublin, have reported others. Hasse says that the cricoid cartilage is the one principally affected. He also speaks of the purulent cyst, external or internal, to the larynx or trachea; both of which observations are verified in the specimen presented to the notice of the Society.

When a student of Prof. Pancoast, Dr. Hall continued: I saw a case of this disease, in which the man was almost moribund; but, upon tracheotomy being performed, the patient rallied, and, after a doubtful period of successive improvements and relapses, recovered his health, although with the loss of a portion of the laryngeal cartilages.

In this case just reported, tracheotomy would also have been performed, had the patient not sank so rapidly.

*Chronic Abscess of the Brain.*—DR. GROSS exhibited a specimen of abscess of the brain sent to him by Dr. W. M. King, one of the resident physicians of St. Joseph's Hospital of this city. The patient was an Englishman, twenty-one years of age, and was admitted on the 8th of November, 1857. He had been a flax-dresser, and consequently led a sedentary life. A short time ago he had emigrated to this country, and during the voyage he had suffered much from the want of the necessaries of life. Soon after his arrival he had repeated epileptic attacks, followed by hemiplegia of the right side, which, however, retained its sensibility. His pulse was small, only forty-five beats a minute; pupils acted readily under the influence of light; the tongue was black; the stomach irritable; and bowels costive. The speech was slow and rather difficult, and he complained of pain through the temples. The treatment consisted in regulating the bowels, applying cold to the head, and administering iodide of potassium and mercury. This course was continued until the 12th of December, when he was able, with the aid of a crutch, to hobble around his room. His appetite had greatly improved, and his pulse had averaged about fifty beats a minute. His articulation, however, was rather worse, and his right arm was perfectly useless. From this time he grew gradually worse; he suffered from constant irritability of the stomach, and, at the end of a week, he had several violent epileptic convulsions. The attack came on in the following manner:—He would fall back apparently lifeless; his extremities were stiff, and he foamed slightly at the mouth; his respiration was feeble, and his pulse was beating at the rate of forty-five a minute. After remaining in this condition about fifteen minutes, he would draw several deep respirations, and gradually revive; but in a few moments another convulsion, more violent than the preceding one, and of much longer duration, would take place. On the 26th of December his appetite still continued good, although he could retain nothing on his stomach; he slept well, but when he awoke was in a stupid condition, with his eyes partially closed. He had ceased to complain of the headache, which had been so troublesome. In a few days he had almost entirely lost the power of deglutition, and, upon assuming the semi-erect posture, he fainted. On the 5th of January the patient had several convulsions, and died quietly early the following morning. On dissection, the brain seemed to be atrophied, and so much softened that a stream of water, when allowed to fall upon it from a slight height, washed away a considerable portion of its substance. In the interior of the left hemisphere there was a large abscess containing about two ounces of fetid pus, and lined by a thick secreting membrane.

*Cancer of the Fundus and Body of the Uterus Perforating the Rectum.*—This specimen, presented by DR. HARRIS, was obtained from the body of a married lady, aged fifty-four years, who was affected with cancerous disease (dating from the time when the first symptom of uterine disturbance made its appearance) for about four years and three months. In early life, and up to the period when the disease commenced, she was remarkably healthy, and for some years anterior to the cessation of her menses, had been of very full habit. At the age of forty-eight she ceased to menstruate, and no bloody discharge came from the uterus during the two succeeding years. A sanguine fluid then commenced to escape from the vagina in small quantity, and this discharge continued without intermission, until the disease was so far advanced that the nature of the fluid became changed by admixture with broken-down cancerous tissue and purulent secretion.

For about two years and five months there was no pain attendant upon the disease; the patient very slowly lost flesh, which was not much remarked by her friends, who supposed it to be due to the effects of age, as she made no complaints of ill health, and called in no medical advice. The first severe pain experienced came on immediately after taking a cold plunge bath; it was felt in the iliac regions, and was of a lancinating character. From this time until a few weeks anterior to death, a period of about twenty-one months, this pain returned daily with a gradually increasing severity, until in a few months it became excruciating, and required a constant resort to anodynes to relieve it. For a long time it returned at a regular hour each day or night; it was occasionally much less acute for a day or two, and sometimes for several days was reduced to a form much more readily to be endured; after which the lancinating character of the paroxysms would return.

Medical advice was first called in about twenty months before death; at which time there was a constant oozing of blood from the uterus, though small in quantity, and a daily paroxysm of pain, as yet not very severe. There was no tenderness of the abdomen upon pressure, the patient being in the habit of forcibly compressing the abdominal walls, and striking herself in the iliac region for the purpose of alleviating her sufferings; and no tumor could be distinguished by the most careful palpation. A vaginal examination, either by the touch or speculum, was not permitted, so that the diagnosis had to be made from the two symptoms mentioned: viz., lancinating, paroxysmal pain, and constant menorrhagia, taken in connection with the fact that the parents of the patient had died of cancerous disease. The malady was pronounced cancer of the uterus, and treated as such.

After being under medical treatment a little more than a year, the patient, by much persuasion, was brought to submit to a per vaginam examination. The uterus was then found enlarged, and its cervix very hard to the touch, but smooth upon its vaginal surface; the whole organ, judging from the size of the neck, was thought to be quadruple its natural dimensions, which could be more readily determined, as the patient had never borne children.

Seven or eight months before death the discharges from the vagina became slightly changed in character, purulent matter being mixed with the bloody fluid, and pus was soon afterwards found in the excrementitious matters from the rectum. The purulent matter escaping from the vagina gradually increased, and became fetid, though seldom so offensive as not to admit of the odor being corrected by the use of chloride of lime or other disinfecting agents.

About four months before death, fecal matter commenced to pass by the vagina, and toward the latter period of life, nearly the whole of the excrement passed through this channel. Frequent urination became necessary to the comfort of the patient, owing to the pressure of the enlarged uterus upon the bladder preventing its distention, though the act was not always readily performed, for upon two occasions it was attended with very severe pain; the fluid was retained for a considerable time. On the last of these occasions (nine weeks before death) the urine was not passed for twenty-four hours, and the distended bladder, by pressing upon the uterus, produced an alarming hemorrhage, after the occurrence of which, a voluntary evacuation took place.

Defecation was painful and exhausting during the latter months of existence. Before perforation of the rectum took place the bowels were generally costive,—four days to a week sometimes elapsing without a passage. No diarrhœa ever occurred except upon one occasion, when it was owing to the patient, unknown to the medical attendant, taking an active cathartic; the attack lasted two days.

During the nine weeks subsequent to the attack of hemorrhage, the patient remained continually in bed; suffered much less pain than formerly, except an occasional paroxysm; lived almost exclusively upon milk-punch; passed large quantities of puriform matter, mixed with fæces, from the vagina; and was troubled much with nausea, and, when she attempted to take any solid food, with vomiting. During the last few days she suffered but little, and died finally from exhaustion.

Autopsy, made sixty hours after death.—Body much emaciated, considering the size of the patient when in full health; but not so much reduced in the extremities, particularly the lower, as is often found in subjects that have died of cancerous affections. The trunk was very thin, and the abdominal parietes flattened, but the opposed portions of the thighs, at their upper portion, still retained a considerable adipose deposit, the skin covering which was red and excoriated from the vaginal discharges. The abdominal walls contained but little fat, and were so much contracted that scarcely any trace of the umbilicus could be discovered. When the abdominal cavity was laid open, the omentum was found to be joined to the fundus of the uterus; and in front of this adhesion the cavity of the womb communicated with the cavity of the abdomen by an irregular aperture, with very soft and rotten edges. In drawing up the omentum to examine more minutely the extent of the adhesion, the adherent portion of the uterus was detached from the remainder of the organ, thus enlarging the opening in the fundus. The body of the omentum presented no abnormal condition, except that it was somewhat more dense and thick than it is usually found. The caput coli was also joined to the uterus, and the vermiform appendage was united by a deposit of lymph to the side of the colon, and was also involved in the connection with the uterus. The large intestine, except the adherent portion, was found healthy from this point to the commencement of the rectum, where were discovered two oval gangrenous perforations near to each other; one large enough to admit the end of the forefinger, the other of smaller dimensions. The liver had undergone fatty degeneration, presenting the most perfect example of this pathological condition I have ever met with. It was very light in color, solid, greased the knife used in cutting into it, and under the microscope showed very large fat-cells. The spleen had undergone no change. The stomach contained between one and two ounces of brownish-yellow bile, and its mucous membrane was much stained by this fluid, but the organ was unaltered by disease. The kidneys were imbedded in fat, and contained adipose deposits in their calyces, but the tissue proper was unaffected. The small intestine and mesenteric glands were healthy.

The uterus was found to be the main seat of disease. It entirely filled the pelvis, with the exception of the space occupied by the contracted rectum and bladder; its fundus was on a level with the superior strait, which accounted for its not having been felt during life. The rectum was more in the median line than usual, and the sigmoid flexure more to the right side. The whole of the pelvic contents were dissected out in mass, and a more minute examination revealed the following:—The ovaries were cancerous, but not larger than natural; the uterus had undergone cancerous degeneration; the os was oval, soft, patulous, and readily admitted the thumb; the cervix was short, very

FIG. 6.

much developed laterally, and had lost its scirrhous condition; the cavity of the neck and body of the organ was greatly enlarged, being capable of containing four or five ounces of fluid, and was filled with a pultaceous mass, composed of broken-down cancerous tissue and fecal matter; the posterior wall of the body of the uterus was united to the anterior of the rectum, and the cavities of the two viscera, communicated by an ulcerated opening an inch wide and three inches long; the non-adherent portion of the rectum was contracted, most probably from want of use, as it was not diseased; the vaginal surface of the cervix was not diseased, and there was no protrusion of fungus through the os; the vagina was found much shorter than natural, and dilated at its uterine extremity to more than two inches in diameter—no cancerous disease existed in its mucous lining. The bladder was united to the anterior wall of the uterus, and was contracted, but the disease had not extended entirely through its walls.

The abdominal cavity contained no effused fluid, pus, or fæces; and, besides the points of adhesion mentioned, there was no other sign of local peritonitis. The perforation at the fundus of the uterus must have been very recent, and most probably occurred after death, by the decomposition of the part, which was very thin and soft,—an event which was favored by the fact that the body was not placed under the preservative influence of ice until twenty-four hours after death. The utero-rectal communication must have existed for at least six months, for as early as that, purulent matter escaped through the rectum.

The soft cancerous matter contained in the cavity of the uterus, when examined under the microscope, exhibited broken-down cells, having nothing characteristic of cancerous disease. The cancer tissue proper was of a medullary character, and easily broke down under pressure; the microscope revealed in it an abundance of oval cancer-cells, with nuclei and nucleoli. This cancer tissue appeared to the naked eye to be infiltrated in the tissue proper of the uterus, constituting a form of disease known as *Infiltrated Medullary Carcinoma.*

This case presents several points of interest which are worthy of our attention, as bearing upon the anticipation and recognition of internal cancers.

1st. The patient was an example of that remarkable robust health so commonly enjoyed by those in whom there exists a strong hereditary predisposition to cancerous disease. Both parents and a half-sister having fallen victims to cancer, her healthy appearance was an additional reason for the anticipation of the same disease; and the first symptoms of uterine disturbance, under such circumstances, would naturally lead to a suspicion of cancer, and greatly assist in making a prognosis of the case.

2d. The medical history of the family of the patient goes to show that cancerous parents are very apt to transmit cancer to their children, but that the seat of the disease in the latter is less influenced by the former than its nature. In the father the disease was seated in the cervical glands; in the mother in the mammæ; in one daughter by a former wife, and in the case here reported, in the uterus. The cervix uteri is the most common seat of cancer, and where there is an hereditary predisposition in a female, this point is most apt to be attacked, no matter what be the seat of disease in the parents. In some rare instances, however, we meet with cases where not only is the disease the same in form, but it occupies the same situation (though an unusual one) as it occupied in the parent. In a patient under my care in 1848, a cancer as large as a small orange occupied the extremity of the nose; the father of the woman died of the same disease, similarly situated. This woman was also remarkably robust previous to the commencement of the disease.

3d. The entire freedom from abdominal tenderness that existed, detracts from the reliability of this symptom as an evidence of the existence of cancer of the uterus.

4th. The inability to detect by palpation the presence of cancer of the body of the uterus, is no evidence that it does not exist, as the prolapsed condition of the womb may render its being felt impossible, even in an emaciated subject.

5th. The absence of pain in the early stages of uterine cancer may readily mislead us in making a diagnosis, where a per vaginam examination cannot be obtained, as menorrhagia may arise from a variety of causes other than the existence of scirrhus. Where there is an evident predisposition to the disease, the return of the menstrual discharge after a long period of cessation is to be regarded as a dangerous symptom, and particularly in those who have previously enjoyed vigorous health.

The specimen of disease before us exhibits what it is rare to find in uterine carcinoma, *i.e.* extensive disorganization, without ulceration of the cervix and vagina. The primary seat of the malady, instead therefore of being the neck, which is almost universal, must have been the body of the uterus, and, judging by the ulceration, that portion of it contiguous to the rectum. The total destruction of the neck of the uterus, upper portion of the vagina, and contiguous walls of the rectum and bladder, is much more common to be met with than perforation of the rectum or bladder, without ulcerative destruction of the cervix. In this, as well as in all the cases of cancer of the abdominal or pelvic viscera I have examined, there were no signs of general peritonitis manifested during life, or found after death. Cancerous union of viscera it is common to meet with, and this is in all probability preceded by a subacute local peritonitis; but general peritoneal inflammation is rare in cancers of the abdominal or pelvic organs, particularly where the uterus is the seat of disease. Another marked feature in uterine cancer is, that with the exception of contiguous viscera, which are diseased by extension from the primary seat, cancer is rarely found in other organs, not even in the mammæ, with which there is such a marked sympathetic connection. Occasionally, in cases of long duration, a deposition of cancerous matter takes place in organs not contiguous. The liver, lungs, and glands of the groin are most apt to be thus affected.

Dr. Gross would ask whether Dr. Harris did not think that the rectum and uterus had become simultaneously affected? There was evidently much disease of the rectum; and it seemed to him not improbable that it had become cancerous at the same time, or shortly after the uterus, and was not simply an extension of the disease of one organ to another.

Dr. Harris believed the case to have been a primary cancer of the uterus extending to the rectum. There were no signs of disturbance of the intestine until the last year of life, while long anterior to this, signs of a morbid state of the uterus existed.

Dr. Woodward directed attention to the fatty degeneration which had here existed. He felt persuaded that in cancerous formations, fatty degeneration of organs, not themselves affected with cancer, very frequently occurred; indeed, almost as frequently as in tubercle. He had especially noticed this in the liver and heart. He also alluded to some recent observers, who had stated that, in malignant growths of the uterus, the muscular fibres increased. He would ask whether this had been noticed in the specimen just described?

Dr. Harris answered, that in the parts examined by him with the microscope no fibres of any kind were perceived; but he had not specially examined those portions of the cancerous mass which stretched into what appeared to be the still healthy structure of the uterus.

*Cysts of the Thyroid Gland.*—Dr. Agnew presented, for Dr. S. W. Gross, a serous cyst of the thyroid gland, with the history and an account of its minute structure. Mary Neff, aged twenty-five, a widow, with one child three years old, came to the Jefferson College Clinic, on the 19th of April, on account of a swelling in the median line of the neck, just above the sternum. She said that her attention was first directed to it by her friends, five weeks previously, when it was the size of a filbert. It rapidly increased, causing a sense of fullness in the throat and slight impediment to respiration and deglutition. Her general health was not good; she occasionally felt sick at the stomach, had no appetite, and now and then vomited. Thinking that this derangement of the system had some connection with the tumor, she applied for its removal. About four days before coming to the clinic, the tumor was painful and the integuments discolored. She was ordered to be leeched, and to take purgative medicine. The tumor being very hard and elastic, fluctuated slightly, and moved with the trachea on deglutition ; a small puncture being made, a few drops of fluid, resembling black tea, escaped. On the 2d of June, Mary was brought before the class, and the cyst was removed by Professor Gross, by an incision in the median line of the neck. It was overlapped by the sterno-hyoid and thyroid muscles, and was pretty firmly connected to the deep cervical fascia and the isthmus of the thyroid gland. It was, however, readily enucleated by the handle of the scalpel. A large branch of the right inferior thyroid artery, which ramified over the surface of the tumor, was ligated ; the blood oozed from many points, and a very small portion of the isthmus was included in a ligature. The wound was left open for about an hour, being kept constantly covered with compresses, wet with cold water. The dressings consisted of four silver sutures, adhesive strips, and a compress. The patient progressed favorably to a cure, the whole of the wound having united in three weeks.

*Anatomical Characters of the Cyst.*—The cyst was of a bluish color, of a globular shape, and about as large as a medium-sized egg. The opening in it made by the exploring needle two weeks before the operation, had not closed. It consisted of an outer connecting cellular coat, a middle fibro-cellular, which could be divided into several very fine layers, and an inner lining membrane, which was smooth and glistening like any of the serous membranes of the body. Its walls received quite an abundant supply of blood-vessels from the surrounding parts, and were thicker in some places than in others. In the lower portion of the cyst there was an opake fibro-albuminous deposit, about three-fourths of an inch in diameter ; but, with this exception, it was either perfectly transparent or translucent.

*Microscopical Appearances of the Lining Membrane.*—This consisted of a structureless membrane, here and there fibrillated, and lined with large, pale, delicate polyhedral cells, having distinct and large oval nuclei, resembling tesselated epithelium. It was coated with altered blood-corpuscles, a large quantity of granules and crystals, for the most part prismatic, but occasionally hexagonal or occurring in masses, resembling the so-called blood-crystals. As seen with the naked eye, there were adhering to it in parts, small portions of coagulated blood, and a few small pedunculated, amber-colored bodies, resembling somewhat in appearance a gelatiniform polypus, and consisting of the same altered blood-corpuscles and crystals. Here and there were cells entirely filled with granules.

The lower portion of the cyst was thickened about one half a line, and contained, between the lining membrane and outer coat, a soft, dirty-yellowish substance, composed of imperfectly-formed cells, embodied in a fibro-albuminous matrix.

*Physical, Chemical, and Microscopical Characters of the Fluid.*—A drop of the greenish-black fluid, which escaped from the puncture, was found to contain shrivelled corpuscles with irregular cell-walls. They were in great abundance; and evidently old and altered, not a single globule being of the normal shape. Upon opening the cyst the fluid was found to be of a dirty brownish-red color, and to amount to six drachms. It had the odor and taste of blood, with a specific gravity of 1·027. It contained a great amount of albumen, as was shown by the action of heat, corrosive sublimate, and nitric acid. After standing fourteen hours, it separated into two parts, the serum constituting about nine-tenths, and the globules the rest of the amount. Upon subjecting a drop of the serum to the microscope, the same altered corpuscles appeared in abundance, as well as a few blood-crystals and cholesterine.

*Remarks.*—Cysts connected with the thyroid body are of rare occurrence when compared with other affections of this gland. During the last two years, I have seen not less than five cases of this affection, three having occurred at the clinic of the Jefferson College; but the one now communicated, is the only one which I have been able to examine minutely. About the same time that the above patient presented herself at the clinic, a German woman, twenty-one years of age, also consulted Prof. Gross for a tumor similar in physical appearances to that of Mary Neff. She attributed its origin to the effects of parturition, it having made its appearance two months after the birth of her first child. In the case of which I have now given an account, it will be seen that the patient had not been confined for three years, nor could she assign any cause whatever for its appearance. It is not at all probable that the tumor had any connection with labor.

In another case, the specimen of which I obtained last winter at Dr. Agnew's anatomical rooms, and which I send with this communication, it will be observed that the cyst is globular, and of nearly the same size as the one which was removed from the patient. When first seen by me, a puncture had been made into it, from which exuded a dark-colored fluid, containing what I supposed to be disintegrated blood. This cyst, also, was connected to the isthmus of the gland at its inferior part, an throughout its whole extent intimately to the substance of the left-lateral lobe. The supply of blood, in this case, came principally from the left superior thyroid artery, a large branch of which ramified over the posterior surface of the cyst. The thyroid plexus of veins was very much enlarged. The gland itself was hypertrophied, which was not the case in the other two subjects, and I have left the arteries supplying the gland, mutilated as they have been in the dissecting-room, in order to show the reciprocal influence of the enlargement of the one upon the other. In this specimen the cyst has become hardened from immersion in alcohol, which prevents an analysis of its contents. From its extensive connections with the gland, extirpation would have been a formidable procedure.

Much diversity of opinion exists in regard to the formation of these tumors; nor shall I weary the Society by presenting them, even with an epitome of the opinions of the principal authorities in pathology concerning this point. It is not always possible to say whether, in their production, the cyst is produced before its contents, and conversely, or, in other words, which was the cause, and which the effect.

In some such as the sebaceous and the true hydatid, we know there is a sac that secretes its contents, which, in their turn, are the cause of its distension; but we cannot with equal certainty trace the origin of some cysts lined by an adventitious serous membrane, which, in most cases, is supposed to be a new formation from a perversion of nutritive action. In regard to the present specimen, I am disposed to attribute its appearance to the formation of an adventitious serous membrane in one of the cells of the areolar tissue in the neighborhood of the isthmus of the thyroid gland. This cell, becoming distended, was finally closed by slight inflammation, and the secreting surface furnished the contained fluid which rapidly increased. The existence of blood in the fluid of the cyst may be attributed to an accidental hemorrhage into its interior during its growth; or, what is more probable, may it not have been a result of perversion of the nutritive functions of the part, in consequence of an accidental inflammation?

DR. DA COSTA presented the following paper on *Cancer of the Pancreas.*—At a former meeting of the Society I exhibited a specimen of primary cancer of the pancreas, and was requested to report more fully on the occurrence of this affection, and on the symptoms by which it is marked. In accordance with this wish, I beg leave to present this paper, accompanied by a table of thirty-seven cases, derived from various sources, and including two brought before the Society.

I have not endeavored to swell the number by instances adduced from the older writers; I have not included in the subjoined table the three cases of Morgagni, the five cited by Bonetus, or the thirty-six observations on scirrhus of the pancreas, which Lieutaud has collected; nor have I referred to the oft-mentioned, but exceedingly unsatisfactory accounts given by Heberden; but I have attempted to bring together the cases of pancreatic disease which have been published by authors still living, or not long deceased, and such as seemed to have been undoubtedly cancer of the organ.

The great difficulty, indeed, in studying cancer of the pancreas is, that, while the older writers have most evidently confounded all chronic alterations of the pancreas under the title of scirrhus, many of the later physicians have taken the ground that cancer does not affect this gland, that all the observations, certainly those of primary cancer, are erroneous, and have brought about a skepticism with reference to the whole subject, which, in connection with the rarity with which the organ is carefully inspected in post-mortem examinations, has tended much to retard our knowledge of its morbid states. Yet there are (leaving out the descriptions of the older writers) a sufficient number of well-authenticated cases of disease of the pancreas on record, not only to prove that the gland is frequently the seat of cancer, but also that, in all probability, cancer is the most common of its chronic affections. These very cases, too, demonstrate that the malignant diseases of the organ are not always, as has been affirmed, secondary, but that cancer may commence in the pancreas and be confined to it, or else extend from it to surrounding textures. (*See cases recorded in the Table.*)

When the pancreas becomes cancerous the disease usually attacks its right extremity. The whole gland may be equally affected, or only the middle portion and the splenic end suffer (*Case* 31;) but this is not frequent. For the most part the cancerous change takes place mainly, if not solely, at the head, the other portions remaining healthy, becoming indurated, or undergoing a fatty degeneration. The disease shows a great tendency to spread to the adjacent lymphatic glands, and a cancer of the pancreas often in reality consists of the transformed head of the organ, so closely blended with these glands, as to have occasioned an apparently uniform tumor of considerable size, which, by pressure, produces obstruction of the ducts leading from the liver, or changes in structure in the surrounding tissues.

Scirrhus and encephaloid are both met with in the pancreas, and run the same course as in other organs. Colloid deposits, too, have been described as occurring. (Dr. Wilks; see Table, *Case* 32.) The natural structures disappear entirely, and the microscope exhibits nothing but abnormal cells, or else the cancer may be infiltrated through the regular gland-tissue.

The form and size of the pancreas are materially modified by the cancerous disease, especially is the size. Enlargement almost invariably occurs, and the organ may exceed three or four times its natural bulk. Duponchel* relates the case of a soldier who died at Cadiz after a long and

* Bulletin de la Societé Med. d'Emulat. Mars, 1824.

obscure disease of the abdomen, and in whom a large tumor, of the size of a child's head, consisting of a brownish matter resembling coagulated blood, and of a broken-down cerebral-like substance, was found occupying the place of the pancreas, of the glandular structure of which not a vestige remained. A mass of similar size occurred in the case of a woman described by Caspar.*

The pancreatic duct often becomes implicated in the disease. Sometimes it remains pervious, but at others it is entirely obliterated. It may be pervious in the diseased mass, or where it opens into the intestine, while at the more healthy portions of the gland it is obliterated. Again, the reverse takes place; it permits the pancreatic secretion to flow, until it reaches the diseased portion of the pancreas, but here and at its mouth it is closed. Cruvelhier† met with what appeared to be a cyst in the pancreas, but which, on closer examination, was proved to be the much-dilated pancreatic duct, the duodenal extremity of which was strongly compressed by a scirrhous degeneration of the head of the pancreas. A still more remarkable case happened a few years ago in the clinical wards of Professor Bamberger.‡ The duct, by pressure at a part of its course, was dilated into a cyst, containing a yellowish-red fluid, of the size of a man's fist. True cysts, however, occur in cancer of the organ; their walls are thin and translucent; their contents may be a bloody serum, (*Case* 19,) or blood mixed with broken down tissue. (Duponchel, *loc. cit.*)

The effects of pancreatic cancer show themselves chiefly on the adjacent organs. Secondary cancers in the brain are not described, nor do the thoracic viscera become often affected. Albers mentions a case in which the lungs were filled with small, yellowish cancerous deposits; Bennett (Clinical Lectures) one, in which gelatinous-looking masses in the lung proved, when microscopically examined, to be cancer; and it is not improbable that the "pulmonary consumption" in Sewall's case (*Case* 3) was cancer of the lung, as it did not come on until a long time after the pancreatic disease was fully developed. The stomach, intestine, and liver, from their proximity to the pancreas, are the organs which suffer most. The *stomach* may be perfectly healthy, or it may be adherent to the pancreatic tumor and thickened, especially near the pyloric extremity. The thickening is due to a simple increase of the normal structure, or to a cancerous deposit in the coats of the organ. The pyloric orifice may be narrowed, and so pressed upon by the tumor as to be nearly obliterated, (*Case* 2.) The mucous membrane of the stomach

---

* Caspar's Wochenschrifft, No. 9; quoted in Canstatt's Jahresbericht, 1844.

† Essai sur l'Anat. Pathol., tome i., p. 286, 1816.

‡ Vol. vi. Virchow's Path. and Therap., p. 667.

is found in a state of softening, or of thickening, but for the most part it is perfectly healthy. The viscus may be much distended, or contracted. Its inner surface has been observed to be covered with a dark slimy fluid, or to contain blood, or even as much as a gill of pus, derived (*Case* 22) from a perforation of its coats, through which the pancreatic mass communicated with the stomach. Such cases of perforation have been several times noticed. The perforation occurs at the seat at which the stomach adheres to the morbid mass; the rupture may be at one, or at several places. (Hasenöhl.)

The *duodenum* presents the same changes as are met with in the stomach. It may be adherent to the pancreas, thickened and contracted, or distended; its calibre may be nearly obliterated by pressure, (see *Case* 23,) its mucous membrane softened, and one or several ulcerations communicating with the pancreas exist in it. The other parts of the intestine generally remain healthy; yet they, too, have been noted to have been greatly contracted, to have been ulcerated, (*Case* 15,) to have contained (*Case* 23) small cancerous masses, or to have been nearly filled with blood. The colon has been observed to be much contracted, and its coats thickened and covered with small patches of lymph, (*Cases* 17 and 18.) There seems, indeed, to be a very great tendency to fibrinous deposits, and to an increase and thickening of the cellular textures of the body, as witnessed in the intestines, and also in a cirrhosed state of the liver.

The *liver* is very variously affected. It remains healthy, becomes the seat of cancerous deposits, or exhibits abnormal changes with reference to size, density, and color. One of the most frequent appearances is to find it enlarged, and of a peculiar greenish hue. Dr. Bright, in an oft-quoted case, describes it as resembling "dark greenstone porphyry." It may be softer than natural in consistency, but is frequently denser, owing, in some instances, to a thickening of its cellular tissue. Well-marked examples of cirrhosis (*Cases* 26 and 34) have also been observed. In common with all the other organs in the body, it is at times pale and devoid of blood, (*Cases* 7 and 29.) The biliary ducts may be normal, or have their calibre greatly increased. The hepatic duct, as well as the cystic and common duct, are at times in some parts nearly obliterated, while in others they are much dilated; or one duct is dilated and the other compressed. Again, both the hepatic and cystic duct may be expanded, and the common duct be barely pervious, (*Case* 9.) In a case described by King,* the hepatic and choledoch duct above the seat of their compression were dilated to the size of the ileum of an infant. Todd† had a young girl under

---

* Medico-Chirurg. Review, 1827, (See Table, *Case* 1.)

† Dublin Hospital Reports, vol. i.

his care, in whom the hepatic and common duct were so distended as to form a distinct swelling in the epigastric region, which was tapped during life, and was found to contain several quarts of bile. The cystic duct alone may be closed; but the duct which most frequently suffers is the common duct. It is evident, however, that the exact spot of its occlusion, or the state above the seat of compression of it, or of the cystic and hepatic duct, will depend much upon the shape and size of the pancreatic tumor.

The gall-bladder, in cases of compression of any of the ducts connected with the biliary function, is enlarged and greatly distended. Its coats have been observed to be much thickened, and its mucous membrane slightly ulcerated, (*Case* 23.) Its contents are a dark, inky bile, or an inodorous, colorless fluid, (*Cases* 1 and 15,) which King (*loc. cit.*) tells us has no resemblance in chemical composition to bile.

The other structures situated in the abdomen do not often become affected in consequence of a pancreatic cancer. The omentum may be implicated in the disease; the spleen remains healthy. The supra-renal capsules were involved in a case described by Dr. Bright.* The diseased mass may press upon the nerves and narrow the aorta, as in a case quoted by Mondière from Portal.† In another instance, the latter author has observed an aneurism to have been produced by the pressure of a scirrhous tumor of the pancreas.

The *age* and *sex* of those suffering from cancer of the pancreas may be seen from the following table of thirty-seven cases:—

| Age. | Males. | Females. |
|---|---|---|
| 14 to 22 | – | 2 |
| 24 to 28 | 2 | 1 |
| 33 to 36 | 3 | – |
| 40 to 46 | 4 | 2 |
| 48 to 58 | 8 | 4 |
| 58 to 68 | 2 | 4 |
| 68 to 78 | – | 2 |
| Not stated | 3 | – |
| | 22 | 15 |

These figures certainly show that cancer of the pancreas conforms, in respect to age, to the general laws of cancerous disease. It will be perceived that the majority of cases occurred after the fortieth year. The youngest (*Case* 21) was a girl 14 years of age, the eldest a woman of 76. Rokitansky has mentioned an instance of the pancreas having been found

* Med-Chirurg. Transactions, vol. xviii., *Case* 7. It may not be without interest to state that in this case no bronzy color of the skin is mentioned.

† Traité de l'Apoplexie.

scirrhous at birth. With reference to sex, the majority of cases are met with in men. Of the fifteen female cases, two occurred in colored women. Dr. Walshe's statements concerning sex do not agree with my deductions. He thinks the disease is more frequent in the female.*

The exact *duration* of the affection it is not possible to ascertain. Like all chronic diseases, its commencement cannot be accurately fixed. It would seem that, although it may last for several years, and occasion prolonged suffering, it may also run a more rapid course. It is, indeed, in not a few of the cases specially noted, that the patient had been, up to a certain time,—not a year before his death,—in excellent health. In several instances, no marked symptoms appeared until four or five months before death, and a case has been reported in which the disease seemingly commenced with acute symptoms, and ran on, in eleven weeks, to a fatal termination, (*Case* 36.) In one patient it was ushered in by jaundice, in another by a febrile attack, (*Case* 7.) In one case it is recorded that it followed a sudden disappearance of tumefaction of the parotid and submaxilliary glands, (*Case* 9,) in another, (*Case* 28,) that it was produced by continual pressure against the stomach. Death usually takes place from gradual exhaustion. But it may occur after hemorrhage, or by the development of cancer in other parts of the body, or with the symptoms of an adynamic fever, (*Case* 8.) The patient mentioned by Dr. Campbell (see *Case* 22) expired suddenly, after a sound like something bursting. The stomach had been perforated, and was found to contain a large quantity of pus.

The *symptoms* of cancer of the pancreas are not always the same; they are mostly produced by the effects of the disease on other organs. The affections of the pancreas themselves give rise to few, if any, special symptoms; to none which are constant.

*Local Signs.*—Amid the local signs, one of the most important is the existence of a swelling, or a tumor. In thirteen cases out of the thirty-seven recorded below, a tumor is specially noticed; in one, there was fulness at the epigastrium; and in one at the left hypochondrium; in one fullness at the epigastrium, with resistance to touch; in one, an indistinct hardness at the pit of the stomach, and in another, at the right side of the abdomen, making eighteen cases in which the pancreas had given rise to perceptible signs of its enlargement. The situation of the tumor is mostly noted as in the epigastric region, or between this and the umbilicus. It may extend into the right hypochondrium, or into the left, or (*Case* 22) into both. It may be fixed or movable, (*Case* 35,) with limits not definable, or capable of being accurately determined by the touch and by percussion. In some

* Walshe on Cancer, p. 321.

instances it is painful on pressure; in others not. In several very interesting cases it was accompanied by pulsation and a blowing sound, and might thus have been readily mistaken for an aneurism. In Dr. Battersby's patient (*Case* 17) there was an apparent systole and diastole; the pulsation ceased in two months, but the bruit and the tumor remained. In the patients of Sandwith, Fletcher, Tessier, and McClurg, (*Cases* 20, 24, 27, and 28,) the pulsation continued as a permanent phenomenon. Both pulsation and blowing sound may be accounted for by the tumor lying across, and compressing the abdominal aorta. In Dr. Battersby's case, however, the blowing sound may have been produced by the deposits which covered the inner coat of the abdominal aorta.

An epigastric tumor of a different nature may be caused by disease of the pancreas, and lead to singular errors of diagnosis. Petit* operated on a case of what he thought to be a strangulated hernia of the stomach or colon. The tumor was soft and compressible, and accompanied by vomiting and hiccough. The operation demonstrated that it was the stomach, pressed forward by an enlarged pancreas; whether cancerous or not, was not determined. In another case already cited, (See *Case* 21,) an epigastric tumor was not the cancerous pancreas itself, but a dilatation of the hepatic and choledoch duct produced by it.

*Pain* is a very constant symptom: it is mentioned in thirty-two out of thirty-seven cases. The seat of the pain is, in most instances, the epigastrium. In twenty of these thirty-two cases it seems to have been there most marked, although it was not always confined to this seat, but extended to the right side, or to the left, or to the back, or to the umbilicus and lower part of the abdomen. In one case it was an intermitting pain confined to the lower part of the abdomen. In two or three others it extended equally over the whole abdomen. In four cases it had its seat of greatest intensity in the back, but in one of these there was also deep-seated epigastric pain, a constant pain in the lower part of the abdomen, and pains extending to the arms. In another case they radiated to the left half of the chest, and to the abdomen. In three cases the pain was mainly felt in the sides, and extended into the back.

The character of the pain is very various. In the majority of the cases it is severe, in some excruciating, and in paroxysms of several days duration. It is, at times, much like colic; or again it is described by the patient as "a deathly distress," (*Case* 29,) or (*Case* 19) as a "hot sensation extending into the back." In some cases it is very slight, more of an undefined uneasiness (*Case* 9) than actual pain. In Andral's patient at La Pitié,† the pains were like blows of a hammer, or like the perforating

---

* Discours sur la Medecine du Cœur: Lyons, 1806.

† Lancette Française, No. 16.

dart of a dagger, and increased at night. The pancreatic tumor was found, on post-mortem examination, to have compressed the nervous plexus which spreads around the abdominal aorta. The pain is not, as a rule, increased by taking food, for this is only noted in very few of the cases, (see 18 and 29;) on the other hand, there are instances in which it is specially stated that it was not. The pain may become duller (*Case* 32) as the disease advances; it may or may not be increased on pressure. It may be suddenly augmented by turning in bed from side to side, (*Case* 14.) In not a few cases is it increased by the erect position, and hence we find patients seeking relief by stooping, and curving their body forward so as to relax the abdominal parietes, (see *Cases* 2, 3, 5, 13, 28.)

*Vomiting* is a symptom, the frequency and importance of which it is difficult to determine, for it is obvious that in those cases in which much disease of the liver or cancer of the stomach were superinduced, it cannot be established in how far the symptom may be placed in connection with the disease of the pancreas. In the thirty-seven cases below noted, vomiting is mentioned in twenty-one; but in two of these it was a transitory phenomenon, lasting a very short time, and occasioned in one, by eating indigestible food. In one case it came on after an attack of hepatitis, which happened seventeen years before any symptoms of pancreatic disease developed themselves; in three others, it occurred in patients in whom considerable disease of the liver and stomach was, after death, detected. Leaving out these six cases, we still find it in fifteen. In nearly all of them it was a late symptom, and in only a few constant. In one patient (*Case* 20) it did not appear until six weeks, and in another not until ten days before death, (*Case* 23,) although in him the pylorus was found greatly contracted. The narrowed state of the pylorus, caused by the pancreatic disease, or the pressure of the tumor on the stomach will explain the vomiting in several instances. In a case mentioned by Dr. Henry Lee,* at the Royal Medico-Chirurgical Society, in which vomiting was among the symptoms, the stomach was perforated by the head of the pancreas, which had produced ulceration by pressure. In another case, (22,) in which ulceration of the stomach occurred, nausea and vomiting became prominent symptoms as the pancreatic tumor increased. The vomited matter consists either of the food that is swallowed (in many cases there is neither nausea nor vomiting until shortly after food be taken) or else (*Case* 18) of a substance like bran and water, of a bilious fluid, (*Case* 9,) of fluid of a glairy character, or of a watery, colorless fluid, (*Cases* 29 and 37;) or, again, the ejection may contain blood, (*Cases* 9 and 16.) The watery fluid that is sometimes discharged may be very

---

* Lancet, 1842.

abundant. It is thought by some to be the pancreatic secretion itself, and not to be derived from the stomach at all; others regard it as an increased salivary flow. The vomited matter is stated in one case, (23,) in which the pylorus was greatly contracted, to have been like coffee-grounds. But the coffee-ground vomit, so often seen in cancer of the stomach, is evidently here but exceptionally met with. In the case kindly communicated to me by Dr. Harris, there was in the vomit a distinct blackish sediment; this was proved to be stove coal, of which the patient was in the habit of consuming daily about half a pint, eating it in the form of cinder.

The condition of the *bowels* is usually that of constipation. In thirty-four cases constipation is mentioned in nineteen; in four the bowels were regular; in three diarrhœa occurred as a late symptom; one patient passed blood and pus by stools; two others, at times blood; in the other cases diarrhœa existed, or alternated with constipation. The fæces are mostly hard, and vary in color according to the presence or absence of the biliary secretion. Hemorrhage into the bowels, which has been observed as occuring in several cases, will explain the black, bloody stools sometimes voided. Dr. Bright has directed attention to the presence of *fatty* stools in cases of pancreatic cancer which he has published (*loc. cit.;*) they were noticed in three cases. But he is far from having affirmed, as subsequent writers wish us to believe, that they are of constant occurrence. He himself speaks of cases of scirrhous pancreas without fatty discharge, and, although he thinks that it is connected with "disease probably malignant of that part of the pancreas which is near to the duodenum, and ulceration of the duodenum itself," he does not, by any means, lay this down as positive, since, at the end of his paper, he suggests that the symptom might be diagnostic "of the *nature* of the diseased action rather than of its seat." Many observers have since brought forward instances of fatty discharges in which no disease of the pancreas existed, and, on the other hand, to the cases of Bright, but few others have been added in which these discharges were associated with affections of this gland.* A desire to bring the physiological teachings of the present day in connection with morbid anatomy may have prompted many to accord so much importance to the occurrence of fatty stools in pancreatic disease. But pathological anatomy seems to contradict the assertion that the pancreatic secretion possesses alone the power to emulsify, and to render the fatty matters fit for absorption. It can certainly

* Dr. Eisemann, Viertel Jahreschrifft für die praktische, Heilk, 1853, (quoted in the Med. Examiner, 1855,) speaks of several cases of pancreatic disease, with abundant fatty discharge, but in the case which came under his own care there was none. In some of the instances quoted the oily evacuations had ceased, although the pancreas was so indurated as to have rendered the performance of its function impossible.

not be the only agent. The cases of Dr. Bright would lead rather to the conclusion that, for fat not to be acted upon, the duodenal secretions must also be vitiated, and the flow of healthy bile interfered with. For in all of them there were also ulcers in the intestine, and the ducts through which the bile flows were compressed or nearly obliterated.

*Jaundice* constitutes, in a large proportion of cases, one of the most prominent symptoms; it is persistent, and resists all treatment. In most instances it does not appear until the pancreas has enlarged considerably, in other words, not until late in the disease; but in a few cases it is noted among the early signs. It usually increases as the disease progresses, and the skin becomes of a deep-yellow, or of a greenish hue, (*Cases* 1 and 36.)

*Dyspeptic symptoms* are a class of symptoms which are found in pancreatic cancer, in a very varying degree. From the vague manner in which the term is made to embrace different states, it is difficult to ascertain the exact nature of these symptoms in the reported cases. They are noted in twenty-five out of thirty-seven cases, some as of early, some as of late occurrence; but of these twenty-five cases there are several in which the signs of indigestion had been evidently present at a time long anterior to the other symptoms of the disease, and probably to the disease itself. *Acid eructations* were troublesome in five cases; in two cases there was much pain after eating; in five cases there was considerable flatulency, incontrollable in one, but not dependent upon taking food, (*Case* 30.) A feeling of weight and oppression at the stomach are noted in three cases; of sinking, relieved by food, in one; of great irritability of the stomach in two. Constant *thirst* is mentioned in six cases, but in one of these diabetes existed.

The *appetite* fluctuates in every conceivable way; it frequently remains good even to the last; it is sometimes capricious, although the patient (*Case* 7) can take a great deal of food; anorexia is noted in seven out of the twenty-five cases in which dyspeptic symptoms are mentioned. *Hiccough* was in two cases (28 and 34) an exceedingly annoying incident. The *tongue* is not often alluded to; from which it may be inferred that it does not often present any peculiarity. It is stated in four cases to have been dry; in two, it was covered with a yellowish coat; in one, with a brown fur; it remained clean throughout in one, and its cleanness and great moisture are especially commented on in two interesting cases (17 and 18.) The *ptyalism*, which sometimes takes place, will give rise to this macerated appearance of the tongue; but, although it may be both very abundant and exceedingly offensive,* the occurrence of this salivary discharge is not frequent, and its importance in diagnosis, therefore, less than some authors state it be.

---

* Mondière. Archives Générales de Medecine, 1836.

*Dropsy* is met with in the advanced stages of pancreatic cancer. It was present in sixteen out of thirty-seven cases; yet, although many of these were complicated with hepatic derangement, in none was it very marked; in most, ascites was present; in some, ascites and anasarca; in one case, marked anasarca of the upper and lower extremities, (*Case* 17,) and only slight ascites; and in another, (27,) œdema of the feet was seen disappearing and reappearing.

*Emaciation* and *debility* are both very striking and constant symptoms. The emaciation is great and progressive. In a case, reported by Sandwith, the patient was so emaciated that the spine could be distinctly traced through the abdominal parietes. Debility usually goes hand in hand with the perceptible loss of flesh, but it may not be as extreme; and, again, it is sometimes prominent among the earlier symptoms. In exceptional cases the loss of flesh is slight, and debility not marked. The *countenance* is usually pallid, and has a distressed look; the features become pinched, and the face is expressive of suffering and anxiety. The skin is sallow, of a bloodless hue, or jaundiced, or more rarely it is straw-colored, (*Case* 28.) The *pulse* is not often noted, when it is, it is stated to have been quicker than in health. A tendency to hemorrhage must also be alluded to; blood was lost from the stomach, bowels, and lungs, in several cases.

The main symptoms, then, of pancreatic cancer, are a tumor in the epigastric region, pain there, or in the back, constipation, progressive emaciation and debility, and obstinate jaundice and occasional vomiting, as the disease advances. The diagnosis is possible, if these symptoms be present, and provided we are able to exclude with certainty the diseases of the stomach and of the liver. I shall not attempt to decide in how far the symptoms may be shared by other chronic affections of the pancreas. Tubercle of that organ is rare, and is associated with tubercle of the lung or of the brain.* Chronic pancreatitis gives rise to many of the same phenomena; but, taking the cases which I have met with in pursuing this inquiry as my standard, I should say that those signs which indicate a tumor, and the symptoms which show its marked growth and pressure upon other organs, are not often present; that pain does not occur to such a marked degree; that the falling off in health is very gradual, and the disease slower of progress, and also that the bowels are not as constipated, but are, on the contrary, more frequently relaxed. It is, however, fair to state, that Dr. Claessen, in a work on Diseases of the Pancreas, (Cologne, 1842,) remarks that constipation in chronic pancreatitis is urgent and enduring.

---

* Würtemberg. Med. Correspond. Blatt.

| Case. | By whom and where reported. | Age and Sex. | Duration. | Local Signs. | Pain. | Vomiting. | State of Bowels. | Jaundice. | Emaciation and Debility. | Dropsy. | Dyspeptic Symptoms. | Other Symptoms, and Remarks. | Post-mortem Appearances. |
|---|---|---|---|---|---|---|---|---|---|---|---|---|---|
| 1 | King. Medico-Chirurg. Review, 1827; from a case under the care of Dupuytren. | 45; male. | Not mentioned; but upwards of four months. | Not mentioned. | Not mentioned. | Not mentioned. | Not mentioned, except that during the day before death, stools of black and bloody appearance passed involuntarily. | Very great (skin of a greenish-yellow color) occurred upwards of four months before death. | Emaciation moderate; debility not mentioned; (excepting as noticed after an operation for cataract; then, also, tendency to syncope, in the perpendicular position.) | None. | Not mentioned. | Cataract of some years' standing. | Pancreas was large; a portion of it forming, with a cluster of scirrhous glands, a tumor, by which the common duct was much compressed, but its mouth remained pervious. The pancreatic duct was free; so was the cystic duct. The hepatic and choledoch duct, above the seat of their compression, were distended to the size of the ileum of an infant, and filled with gas. The gall-bladder, besides gas, contained an inodorous, colorless fluid, which had no resemblance, in chemical composition, to bile. Liver was large and green. Large intestines contained coagulated blood. |
| 2 | Sewall. Med. and Physical Journal, vol. xxxi. p. 96. | 27; male. | About one year. | Tumor in epigastrium. | Severe, deep-seated, epigastric pain, increased by the erect position; hence patient always in a curved position of the body. | Almost constant. | Constipated. | Not mentioned. | Both great. | Very slight ascites; (post-mortem.) | Acid eructations; great irritability of stomach. | Disease preceded by tumefaction of parotid and submaxillary glands, which suddenly subsided. | Pancreas was nearly three times its natural size; hard throughout, irregular, and unyielding. Its right extremity pressed firmly on the duodenum, and on the small extremity of stomach, thereby nearly obliterating the pyloric orifice. Other organs healthy. |
| 3 | Sewall. *Ib.* | "A young man." | A few years. | Not mentioned. | Deep-seated, epigastric pain, increased by the erect position; hence always a curved position. | Present. | Not mentioned. | Not mentioned. | Great emaciation; debility not noted. | Slight ascites; no œdema. | Great acidity; no food except milk could be retained on stomach, yet appetite remained good. | A pulmonary consumption came on two months before death. | Pancreas—only one of the abdominal viscera, which was diseased, was enlarged, and scirrhous, particularly its right extremity, which embraced the duodenum, and pressed so firmly on the pylorus that its orifice would scarcely admit of the introduction of a common-sized catheter. Stomach and intestines were greatly contracted. Liver healthy. Lungs hard and unyielding, and in many places ulcerated, and affected with tubercle. |

| | | | | | | | | | | | | | |
|---|---|---|---|---|---|---|---|---|---|---|---|---|---|
| 4 | Martland. Edinb. Med. and Surg. Journal, 1825. | 60; female. | Six months. | Hard tumor, about the size of the palm of the hand, at scrobiculus cordis and right side, below the margin of the ribs; very painful on pressure, and enlarging toward the end of the complaint. | In and about the region of the tumor. | Not mentioned. | Regular; stools very white. | Most intense; came on a few weeks after first manifestations of the disease. | Great and progressive debility, and extreme emaciation. | Not mentioned. | Anorexia; great thirst; furred and dry tongue. | Pulse between 80 and 100. | Pancreas. The head formed a scirrhous tumor about the size of a hen's egg. In this tumor was lodged the ductus communis, which was almost impervious, and was still more obstructed by a small calculus. The cystic and hepatic ducts were considerably dilated. Gall-bladder much enlarged. Liver studded with small, distinct tubera, which were confluent opposite the gall-bladder. |
| 5 | Abernethy. In a lecture delivered at St. Bartholomew's Hospital. Lancet, April 21st, 1827. | "A man of advanced age." | Not mentioned. | None. | Pain in the epigastric region, gradually extending, and increased by pressure, by erect posture; hence patient constantly stooping forward. He had also to be propped up in bed, to lessen pressure. | Not mentioned. | Constipated. | None; (countenance had a distressed appearance, even from commencement, but was never jaundiced.) | Increasing debility. | Not mentioned. | Pain after eating. | | Pancreas in a state of complete disorganization and ulceration from end to end. State of other organs not mentioned. |
| 6 | Abercrombie. "Diseases of the Stomach," p. 412. Am. ed. | 56; male. | Two years. | Not mentioned. | Pain in the left hypochondrium, extending into back. | None. | Regular. | Occurred only a few weeks before death. | Both present; died gradually exhausted. | Not mentioned. | Symptoms of indigestion present. | | Pancreas, in parts, hard; in others soft, and composed of yellowish and white matter. Mass attached to spine. Liver enlarged and soft. Other organs healthy. |
| 7 | Abercrombie. *Ib.* | 35; male. | Eighteen months. | Not mentioned. | Undefined uneasiness in epigastric region. | As a late and transitory symptom. | Sometimes constipation, at others diarrhœa. | None; (countenance remarkably pale.) | Both present to a marked degree, and progressive. | Not mentioned. | Present; appetite capricious; took a good deal of food. | Strong action of heart; throbbing in head; disease commenced with a febrile attack; frequent perspirations at night. | Pancreas considerably enlarged, and of nearly cartilaginous hardness, except some spots, which were soft, with the appearance of medullary sarcoma. Pylorus thicker than normal, and adherent to pancreas. Other organs healthy, but very devoid of blood. |

| Case | By whom and where reported. | Age and Sex. | Duration. | Local Signs. | Pain. | Vomiting. | State of Bowels. | Jaundice. | Emaciation and Debility. | Dropsy. | Dyspeptic Symptoms. | Other Symptoms, and Remarks. | Post-mortem Appearances. |
|---|---|---|---|---|---|---|---|---|---|---|---|---|---|
| 8 | Andral. Archives Générales de Medicine, 1831; or *Lancette franc.*, T. Y. No. 216. | 54; female. | Four months; previous health good. | Fullness in left hypochondrium, but no tumor perceptible. | Intense pains in dorsal region, extending to the left half of the chest, or through abdominal region; more frequent at night, lasting from several hours to several days at the time. | Not mentioned. | Diarrhœa as a very late symptom. | None. | Not mentioned; (face pale, expressive of suffering.) | None. | Extreme disgust for taking food; tongue had a yellowish coat. | Insomnia; febrile signs; patient died with all the symptoms of an adynamic fever. | Pancreas enormously enlarged, and transformed into a tumor, which seemed a combination of scirrhus, encephaloid, and tubercle. (?) This mass compressed the aorta, and the plexus of nerves which surrounds it. Other organs healthy. A sanguineous effusion into the pericardium. |
| 9 | Becourt. Quoted by Andral, Pathol. Interne. Tome ii. p. 283. | 45; male. | Four months. | Pain on pressure at a small point between umbilicus and curvature of stomach; heat over stomach. | Above umbilicus; abdominal cramps; at times violent pains over stomach, extending over whole abdomen. | Nausea; also vomiting, in last two months, bilious, sometimes sanguinolent ejections. | Costive; hard stools, at times white. | Occurred early, and became intense; slight jaundice, with feeling of oppression at the epigastrium, were indeed the first symptoms. | Great debility; also emaciation. | Slight ascites. | Great thirst; appetite good; cardialgia flatulency. | At times passive hemorrhage. | Pancreas—head scirrhous, rest converted into fat; liver of an olive-color, and containing a few cancerous spots. Gall-bladder distended, containing very dark bile; hepatic and cystic ducts enlarged; common duct very much compressed, and barely pervious. Stomach healthy. |
| 10 | Percival. (Transact. of College of Physic., Ireland,) Vol. ii. p. 132. | "Middle aged man." | Three months. | Epigastrium distended; tumor felt protruding from middle. | Not mentioned. | Bilious vomiting. | Blood and pus passed by stools. | Present. | Emaciation and much debility. | Anasarca toward the end. | | "Disordered secret. of urine." | Liver "much diseased." Pancreas scirrhous, contained a considerable abscess. Ductus com. choled. closed, in the parts adjacent to the pancreas. Gall-bladder full; cystic duct pervious. |

| | | | | | | | | | | | | | |
|---|---|---|---|---|---|---|---|---|---|---|---|---|---|
| 11 | Dr. Bright's Cases. Vol. xviii. Med.-Chir. Transact., 1833. Case I. | 49; male. | One year. | None. | Pain in loins. | Not mentioned. | Stools copious and light-colored; for the last two months fæces contained a yellowish, fatty matter, and bowels were much relaxed. | Present; commenced six months after first symptoms of disease. | Great debility, and emaciation, continually increasing. | Slight ascites; legs very slightly œdematous. | Great thirst and appetite. | Marked enlargement of liver; frequent urination; diabetes; acute pleurisy two weeks before death. | Pancreas hard and cartilaginous to the touch; of a bright-yellow color; its head formed, with the surrounding glands, a hard, globular mass; at junction of pancreas with duodenum ulcers had taken place. Liver resembled dark greenstone porphyry, and contained hard, circumscribed masses; its ducts were enlarged; common duct dilated, but terminated by a *cul-de-sac* in diseased part of pancreas. Signs of jaundice pervaded many structures; serum olive-colored; coating of fibrine on pleura. |
| 12 | Dr. Bright. Case II. *Ib.* | 50; female. | Not ascertained; certainly not less than seven or eight months, perhaps some years. | None. | No pain on pressure; some pain at lower part of abdomen, relieved by pressure, and occurring only at intervals. | Retching and vomiting for seventeen years; increased much within the seven months before death. | Rather costive; evacuations whitish; a few dark motions like pitch; a week before death large coagula of blood; while under observation for three months, fatty matter noticed in her dejections. | Very great; gradually increasing; did not occur as a permanent symptom until four or five months before death. | Progressive and great debility; general emaciation, cheeks much sunken, but some fat on abdomen. | None. | Not very marked. | Good health until seven or eight months before death. Seventeen years ago severe hepatitis; she became very drowsy during the last days of her life. | Pancreas hard and cartilaginous; its head enlarged and glued to duodenum, and communicating through an ulcerated spot with this. Common gall-duct pervious, but evidently had been compressed; biliary ducts distended. Liver cancerous. Lungs healthy, but firmly bound down posteriorly by strong, adhesive bands. |

| Case | By whom and where reported. | Age and Sex. | Duration. | Local Signs. | Pain. | Vomiting. | State of Bowels. | Jaundice. | Emaciation and Debility. | Dropsy. | Dyspeptic Symptoms. | Other Symptoms, and Remarks. | Post-mortem Appearances. |
|---|---|---|---|---|---|---|---|---|---|---|---|---|---|
| 13 | Dr. Bright. *Ib.* Case III. | 21; female. | Decided illness for two months. | Indistinct hardness on right side of abdomen. | No pain mentioned. | Not mentioned. | Rather bound, but subsequently evacuations were copious, fatty, and thin. | Slight; increased towards close of life. | Emaciation not great, but great debility; restlessness. | Anasarca; also some ascites. | Not mentioned. | Out of health for two years; slight cough; preferred lying in a raised position. | Pancreas—hard mass near its head; another near the spleen; intervening portion seemed more healthy; masses of yellow color. Ulcers in the intestine; some ulcers communicating with glands in the meso-colon; mesenteric glands and supra-renal capsules diseased; also, bronchial glands; slight deposit of round size at apex of lung. Liver enlarged, of a dark-olive color; hepatic and cystic duct enlarged, but common duct becoming much constricted before entering duodenum. |
| 14 | Dr. Bright. *Ib.* Case VI. | 76; female. | Thirteen months; good health before attack. | None. | Severe pain in the sides, extending to the back; pain also immediately under right mamma; acute pain in turning from left to right side; pain in the side became very fixed. | Vomiting not mentioned; nausea as a late symptom. | Constipated at the latter portion of the disease; clay-colored stools. | Jaundice only latterly present. | Great and increasing lassitude and debility. | Among the late symptoms anasarca; also some ascites. | Weight and distension of stomach. | Palpitation of the heart. | Pancreas large and in a scirrhous condition, involving the ductus choledochus in the diseased structure. The common duct was dilated up to its termination, where it was found completely obliterated; near the duodenum it formed a complete *cul-de-sac*. Liver small, but gorged with bile. Gall-bladder enormously distended. Duodenum thickened, and somewhat contracted. |

| | | | | | | | | | | | | |
|---|---|---|---|---|---|---|---|---|---|---|---|---|
| 15 | Dr. Bright. *Ib.* Case VII. | 35; male. | Thirteen months. | Tumor above umbilicus, reaching not quite to pit of stomach. | Not mentioned. | Not mentioned. | Constipated; stools became, subsequently, clay-colored and yeast-like; a few days before death very dark. | As a late symptom. | Both present to a marked degree; at first greater debility than emaciation. | Not present. | Not mentioned. | A tumor below umbilicus. | Pancreas—malignant disease towards its middle; its splenic and hepatic heads not infiltrated; its duct pervious. Its middle portion was involved and continuous with a large, movable mass, connected with the lower tumor seen during life. This tumor consisted of masses, which surrounded the aorta and iliacs, and, passing up the spine, involved the pancreas and renal capsules. Upper tumor was a movable mass in the omentum. A few scirrhous tubera in liver. Gall-bladder distended. Orifice of cystic duct very nearly closed; hepatic duct and common duct both somewhat contracted; colorless fluid in gall-bladder and cystic duct. Small, malignant tumor attached to surface of the heart. Pancreatic duct seemed obstructed in healthy part of pancreas; in other parts pervious. |
| 16 | Dr. Bright. *Ib.* Case VIII. | 43; male. | Ten months. | Indistinct hardness at pit of stomach. | At pit of stomach. | Towards end of life; severe vomiting of a dark-colored fluid shortly before death. | Relaxed at first; clay-colored stools, then varying in color. | Jaundice appearing suddenly and early; became persistent. | Both markedly present; debility as an early symptom. | Not present. | Flatulency. | Appetite unusually great; itching over body; frequent drowsiness; tendency to hemorrhage. | Pancreas not enlarged, but its head formed a large yellow mass, with neighboring absorbent gland; pancreatic duct greatly enlarged. Liver full of yellow spots, of varying size; liver enlarged; all the ducts involved in cancerous masses; hepatic duct enlarged, and filled with colorless fluid. Stomach full of dark, grumous fluid. Serum in chest of dark-yellow color. Ulcer in duodenum. |

| Case | By whom and where reported. | Age and Sex. | Duration. | Local Signs. | Pain. | Vomiting. | State of Bowels. | Jaundice. | Emaciation and Debility. | Dropsy. | Dyspeptic Symptoms. | Other Symptoms, and Remarks. | Post-mortem Appearances. |
|---|---|---|---|---|---|---|---|---|---|---|---|---|---|
| 17 | Dr. Battersby. Dublin Medical Journal, Vol. xxv. 1844. Case I. | About 58; female. | Sick twenty-five months; disease marked for thirteen months. | Deep-seated pulsating tumor in epigastrium, having a well-marked *bruit de soufflet;* the pulsation ceased in two months, but the bruit and the tumor remained; fullness in epigastrium. | Severe pains in back, extending to arms; then uneasiness and deep-seated pain in the epigastrium, increased by pressure; also, constant pain in the lower part of abdomen. | None. | Very sluggish; passages attended with violent straining and intense distress; fæces generally watery, deficient in bile. | Present, but not to a great degree. | Both present; emaciation extreme. | Slight ascites; very marked anasarca of upper and lower extremities, which increased much at later portion of disease. | Eructations; appetite nearly gone, but this not until last month of disease; no thirst; tongue pale and clean. | Ptyalism; mouth always full of saliva; dysphagia. | Pancreas enlarged and hard throughout; every trace of its natural structure had disappeared. At its lower edge existed a thin cyst, about the size of a walnut. Duodenum extremely contracted, and adherent to pancreas; pancreatic duct was pervious for about an inch only from the duodenum. Liver small, of a dark-gray color, and dense; owing, apparently, to a thickening of its cellular tissue. The common duct and hepatic duct were not interrupted; colon and cardiac orifice of stomach much contracted; cellular tissue increased and hard. Mesenteric vessels and nerves involved in the scirrhous mass; gastro-hepatic omentum dense, hard, and thickened; aorta diseased by deposits in its entire course through the abdomen. |
| 18 | Dr. Battersby. *Ib.* Case II. | 24; male. | Four years. | None. | Severe pain in stomach, coming on generally after meals; subsided after vomiting; sometimes appearing in the middle of the night. | Present; sometimes of dark fluid, sometimes like bran and water. | Severe constipation; diarrhœa at end of life. | Not mentioned; (skin sallow.) | Emaciation; features pinched; debility not mentioned. | Ascites and anasarca, both as late symptoms. | | Cleanness and great moisture of tongue. | Pancreas dense and cartilaginous; confounded somewhat with surrounding structures. Liver healthy. Stomach and intestines distended. The sub-mucous coat of ileum and colon thickened; also covered with small patches of closely-adhering lymph. |

| | | | | | | | | | | | | | |
|---|---|---|---|---|---|---|---|---|---|---|---|---|---|
| 19 | Crompton. Birmingham Path. Society; in Prov. Med. Journal, Dec. 1842. | 60; male. | Upwards of two years. | None. | Constant pain below the ensiform cartilage; sometimes "a hot sensation," sometimes pain extending into the back. | None, until last month, then only for two days, after eating indigestible food. | Tolerably regular; at times somewhat relaxed; dejections of good color. | Slight and not permanent. | Extreme and progressive emaciation; (a peculiar pallid appearance of countenance noted.) | None. | Appetite irregular; suffered more after a full meal; mouth generally clammy; tongue constantly covered with a brown fur at the base, and down the centre. | Pulse somewhat quick; skin dry and harsh. | Pancreas hard as cartilage; its left side distended by a large cyst, containing a bloody fluid. Many of the mesenteric glands enlarged and hardened. Liver small; scirrhous tubercles scattered through its substance; cystic duct obliterated by a deposit. A few calcareous deposits in lungs. Other organs healthy. |
| 20 | Sandwith. Ed. Med. and Surg. Journ. Vol. xvi. p. 380. | 67; female. | Not mentioned. | Pulsation, left side, below cartilage of false ribs. | Continual pain in epigastrium, extending to hypochondrium; at times most intense; increased on pressure. | Not until six weeks before death, then very constant and distressing; everything she swallowed was rejected. | Costive. | None. (Complexion sallow.) | Both; eyes had a peculiar expression of anxiety; emaciation was extreme; spine could be traced through abdominal parietes. | Not mentioned. | Loss of appetite. | Great agitation, tore the bedclothes, etc. | Pancreas presented usual signs of scirrhus. Stomach erythematous. Splenic artery imbedded in scirrhous matter. |
| 21 | Todd. Dublin Hospital Reports. Vol. i. | 14; female. | Some months. | Tense swelling in epigastric region, extending to right hypochondrium; it was tapped, and a greenish fluid escaped. | In epigastrium; increased on pressure; at times severe and very acute. | Not mentioned. | Not mentioned. | Deep orange-colored skin. | Great debility and emaciation. | Ascites and anasarca. | Present. | Spasms and convulsions; had had, for a long time, pains in the abdomen; development of disease followed; a fever, with relapses. | Pancreas; head and glands around it converted into a hard, solid mass; its duct obliterated. Stomach somewhat thickened. Liver healthy; cystic duct dilated, but at its juncture with hepatic it was impervious; remaining portion natural. |

| Case | By whom and where reported. | Age and Sex. | Duration. | Local Signs. | Pain. | Vomiting. | State of Bowels. | Jaundice. | Emaciation and Debility. | Dropsy. | Dyspeptic Symptoms. | Other Symptoms, and Remarks. | Post-mortem Appearances. |
|---|---|---|---|---|---|---|---|---|---|---|---|---|---|
| 22 | Henry F. Campbell. South. Med. and Surgical Journal. Vol. v. 1849. | 72; female. | Not mentioned. | Tumor like large orange in epigastrium, extending into both hypochondriac regions. | Pain in epigastrium; two months previous to death became very distressing. | Nausea and vomiting as tumor increased. | Not mentioned. | Not mentioned. | Both to a high degree. | None. | Present. | Expired suddenly, after sound like something bursting. | Pancreas much enlarged; altered in structure, excepting at left extremity; tumor at the right extremity, with pus on surface, and ruptured entrance, which communicated with a cavity in its interior, and with a rupture in the rear of the stomach. This viscus, containing a gill of pus, was softened towards pyloric extremity. At the greater extremity thickening of its coats. Duodenum, near pancreas, was softened. Liver small, and very dense, of darker hue. Gall-bladder much distended; cystic duct distended; duct com. choled. occluded by tumefaction and induration of duodenum. Wursungian duct only seen at left extremity, and here its calibre obliterated. |
| 23 | Dr. Greene. Dublin Journal of Medical Science. Vol. xxv. 1844. | Male; age not stated. | Nine months. | None, save fullness in epigastrium. | Pain in epigastric region, and over umbilicus, sometimes like colic. | None, except ten days before death, then coffee-ground matter. | Constipated. | Jaundice intense. | Not mentioned. | None. | Anorexia; thirst; acid eructations; signs of indigestion were the earliest symptoms. | A week before his death delirium; died in that state. | Pancreas.—Its head was bound down, with the ascending and descending colon, into a cancerous mass, with which the duodenum was also connected; cystic and hepatic duct obstructed by the malignant growth. Mucous coat of gall-bladder ulcerated in several spots. Cancerous masses in several parts of the small intestines. Stomach dilated; pylorus hard, thick, and firm, and its calibre greatly contracted. |

| | | | | | | | | | | | | | |
|---|---|---|---|---|---|---|---|---|---|---|---|---|---|
| 24 | Dr. Fletcher. Birmingham Path. Society: Jan. 20th, 1844. Prov. Med. Journal. | 52; female. | Not mentioned; (under treatment for two months.) | Hardness and increased space of dullness, extending from the right hypochondrium into the epigastrium; pulsations felt there and in the left hypogastric region; and a distinct *bruit de soufflet* attended each impulse when the patient was in the recumbent position. | Intense, excruciating pain in region of stomach; increased on pressure; pain extended to right hypochondrium, and down to the umbilicus. | Vomiting generally in about half an hour after taking food; constant nausea. | Costive; scanty evacuations twice or three times daily in an advanced stage of the disease. | Not mentioned. | Extreme emaciation. | Ascites; bowels above very tympanitic. | Irritable stomach; great thirst; tongue dry and red. | The whole surface of abdomen was tender; pulse small and feeble, 120 per minute. | Pancreas.—Entire organ carcinomatous; its head very much enlarged, wrapping around the duodenum, and inclosing that intestine in its diseased structure, so as to produce a stricture just below the pylorus. Liver studded with carcinomatous tubercles; other organs were healthy. |
| 25 | Albers. Rheim. Corresp. Blatt, 1843; or, Canstatt's Jahresbericht, 1849. Vol. ii. | 50; male. | Upwards of one year; (previously in good health.) | Fullness of epigastric region; a tumor with an irregular surface felt there, and in the right hypochondriac region; and a pear-shaped, movable body between twelfth rib and the anterior superior spinous process of the ileum. | Above umbilicus, and extending to right hypochondrium; subsequently pain in the left side, extending to ensiform cartilage, and to umbilicus; also, dorsal pain; pain not constant; also, pain over spinous processes of all lumbar vertebræ; at least on pressure; (these vertebræ were very prominent. | As a late symptom; matter vomited; yellowish-green, then dark matter. | Soft, white stools; very late in the disease black stools. | Present, and increased; occurred as an early symptom. | Present. | Not mentioned. | Eructations of a bitter fluid; also, clear, yellowish, green, and acid fluid expectorated; appetite good; very fetid breath. | Dullness on percussion on left side of chest; disposition to lie on the back; salivation for three months, after taking ℈ij. of calomel; urine dark color, containing cholesterine; passed with some difficulty. | Pancreas.—Its head hard and degenerated, forming a yellowish tumor, which, microscopically examined, showed irregular cells, with several nuclei. Similar cells are seen in the small, yellowish deposits in the left lung, and in the liver; pancreatic duct pervious, and could be traced to the middle of the gland. Gall-bladder much dilated. The entrance of the common duct could not be found. Pancreas adherent to duodenum, and at seat of adhesion an ulcer in the latter. |

| Case | By whom and where reported. | Age and Sex. | Duration. | Local Signs. | Pain. | Vomiting. | State of Bowels. | Jaundice. | Emaciation and Debility. | Dropsy. | Dyspeptic Symptoms. | Other Symptoms and Remarks. | Post-mortem Appearances. |
|---|---|---|---|---|---|---|---|---|---|---|---|---|---|
| 26 | Caspar. Caspar's Wochenschrifft, No. 9; quoted in Canstatt's Bericht, 1844; 3. | 40; female. | Not mentioned. | Tumor could be felt, whose edge extended along median line to umbilicus. | Not mentioned. | Present mainly after taking food. | At times stools containing dark blood | Present. | Present. | Ascites as disease advanced. | None. | Vomiting commenced after delivery. | Pancreas, the size of a child's head, and in a state of scirrhous degeneration; cirrhosis of the liver; gall-bladder much distended. |
| 27 | Tessier, Journal de Medic. de Lyon, Nov., 1847. | 33; male. | Not mentioned; (appeared, when first seen, in tolerably good health.) | Hard, pulsating tumor extending from ensiform cartilage to umbilicus; abdomen prominent. In the last stages abdomen constantly rising, and simultaneously so with pulse, each movement accompanied by a sound audible some distance from patient, (glou-glou.) | Present; when the œdema appeared there was violent pain extending to the feet. | Not mentioned. | Constipation. | Doubtful; pale-yellowish complexion. | Not mentioned; but great debility is noted as perceived suddenly two days before death. | Œdema of feet, disappearing and reappearing. | Not mentioned. | Pulse became small; face altered; extremeties cold two days before death. | Pancreas.—Whole of the organ converted into a cancerous mass, which compressed the aorta; a few mesenteric glands around the tumor cancerous. In the pancreas a few softened spots. Other organs were healthy, except the stomach, which was much dilated and filled with fluids. (This supposed to be the cause of the gurgling "glou-glou" sound.) |

| | | | | | | | | | | | | |
|---|---|---|---|---|---|---|---|---|---|---|---|---|
| 28 | J. R. McClurg. Medical Examiner, Phil., 1851. | 50; male. | Eight months. | Fullness in epigastric region, and to the touch the feel of a thickened condition; decided pulsation in the epigastric region; great tenderness there on pressure. | Pain commenced early, and was constant and very intense; sometimes most in the region of the stomach, then in the left side, or in the back; no pain in the right side, excepting a month or two before death. | None. | Costive. | None; (skin dry, of a straw color; countenance had a wild, anxious look.) | Progressive debility and emaciation. | Ascites and anasarca four weeks before death. | Anorexia; feeling of oppression at epigastrium; tongue covered with a thick, yellow coat. | Great restlessness; patient unable to lie in bed; also inability to stand erect; bent forward so as to relax abdominal muscles; at one time fever; pulse, 90. In the last month of the disease hiccough became an almost constant symptom. The patient thought his disease had been produced by having carried, two years previous to his last sickness, a load of coal in a tub, which pressed hard against the stomach, giving him pain at the time, and ever after some uneasiness or a tired feeling at the epigastrium. | Pancreas.—The whole of the organ was converted into a cancerous mass, which also embraced the smaller curvature of the stomach, surrounded the solar plexus, the aorta, and accompanying vessels, and adhered to the diaphragm, the liver, the arch of the colon, and omentum. Liver was enlarged, hard, and "tuburculated;" gall-bladder healthy. |

| Case | By whom and where reported. | Age and Sex. | Duration. | Local Signs. | Pain. | Vomiting. | State of Bowels. | Jaundice. | Emaciation and Debility. | Dropsy. | Dyspeptic Symptoms. | Other Symptoms, and Remarks. | Post-mortem Appearances. |
|---|---|---|---|---|---|---|---|---|---|---|---|---|---|
| 29 | Dr. Kneeland. New York Journal of Medicine. Vol. xi. 1853. | 64; male. | About eighteen months. | Hard tumefaction in epigastric region; not tender on pressure. | "Constant deathly distress," confined principally to epigastric region; increased by nourishment; suffering, indeed, much greater when he did not reject whatever he swallowed. | Present, and as an early symptom; matter vomited colorless; of a saltish taste. | Constipated. | No jaundice. | Extreme emaciation; (skin of a dingy, pale, bloodless hue.) | Not mentioned. | Continual vomiting of food; occasional vomiting and sinking sensation about the stomach were very early symptoms. | | Pancreas enlarged and hard; adherent to stomach; converted, with the portion where it was adherent, into one mass of scirrhus, of a uniformly dull or yellowish-white color, and of a homogeneous structure. Liver, spleen, and kidneys anæmic in appearance. The mucous membrane of the stomach had retained its normal structure; the viscus was much contracted. |
| 30 | John S. Antrum. Association Med. Journal, 1855. | 52; female. | "Several years." | Indistinct fullness in epigastric region, in the later stages; also, indistinct dullness on percussion; at one time a circumscribed spot, an inch square, below the edge of the liver, tender on deep pressure, was noticed. | Continuous epigastric pain, as a very early symptom; at first relieved by change of air and tonics; later more severe, and extending to the right groin, and to the back. | Present, but rarely, and only as the disease advanced, and then appeared to arise from distension by wind; never a black appearance of vomit. | Rather irregular and irritable; stools natural, except when deficient in bile; nothing like fat passed, not even when cod-liver oil was taken. | Never marked; skin gradually became slightly yellow and muddy. | Increasing debility and emaciation; had had attacks of debility for several years. | Not mentioned. | None. Food readily digested; uncontrollable flatulence, but not dependent on taking food; pain in the earlier stages relieved by taking food; seemed more a sensation of exhaustion. | Abdomen became, in the last stages, tympanitic; right thigh became swollen, and femoral vein tender in several parts of its course. Death took place from inanition. | Pancreas.—Its head enlarged to the size of a goose egg, by a cancerous deposit in its tissue. Similar deposits in the smaller end, and in neighboring lymphatic glands. Pancreas not adherent. Pyloric end of stomach, near pancreas, thickened by a deposit of cancer, apparently colloid; deposit ceased abruptly at the pylorus; dark, black, slimy mucus, as is often ejected in cases of cancer of the stomach, covered the pylorus. Other viscera, as far as examined, healthy. |
| 31 | Haldane. Month. Journal of Med. Science, Edinb., 1854. | Male age not stated. | Seven months or upwards. | None. | Not mentioned. | Not mentioned. | Costive; clay-colored stools during jaundice; previously dark and scybalous; not fatty. | Intense, but occurring as a late symptom. | Present, and both continually increasing. | No anasarca; but ascites, with much tympanitis. | For seven months. | Gradually, before death, sank into asthenic coma. | Pancreas cancerous, (by microscope) but disease was not at the head of the organ, which was healthy. Retrograde cancer-spots in liver, and in the mesentery. Gall-bladder much distended; ductus communis compressed, and involved in the disease. |

| | | | | | | | | | | | | | |
|---|---|---|---|---|---|---|---|---|---|---|---|---|---|
| 32 | Dr. Wilks. Transactions of the Pathol. Society of London, Vol. vi. 1855. | 56; male. | Six months. | Abdomen felt rigid in the last stages of the disease, but no tumor perceptible. | In middle of back and in abdomen, varying in intensity; very severe at first; became dull, and lessened much as disease advanced. | None. | Obstinately constipated, but stools throughout normal. | None. | Emaciation steady and progressive; became extreme; debility not specially mentioned. | Slight ascites, as a late symptom. | Appetite bad, but not capricious. | Patient, when first seen, did not appear very ill. | Pancreas changed into a cancerous mass; in part fibrous and hard, in part gelatiniform. The head still retained some healthy structure. The duct, close to the duodenum, was pervious, but quite impervious when running through the diseased gland. The pancreas was closely adherent to the duodenum and stomach. A few of the gastric absorbent glands were partly infiltrated with morbid matter, but they were not connected with the diseased pancreas. The omentum was drawn up, and converted into a hard cancer. In it, and in the pancreas, were found well-marked cancer-cells. No cancer existed in other parts of the body. |
| 33 | Dr. Da Costa. Proceed. of Path. Society of Philadelphia, p. 8; or, North Am. Med.-Chir. Review, January, 1858. | 45; male. | Seven months. | None. | Pain across epigastrium, but not severe; extending to back. | Occasional, and not at commencement of disease. | Constipated; dejections are clay-colored, not fatty. | Very marked; occurred rather early in the disease, and continually increased. | Both present, but neither to a marked degree. | None. | Very slight symptoms of indigestion. | Hemorrhage from the lungs. | Pancreas enlarged; its head and surrounding glands converted into a hard tumor, which, microscopically examined, proved to be a cancer; middle portion in a state of fatty degeneration. Pancreatic duct pervious. Liver enlarged, green and mottled, with irregular and large pigment masses in its structure; hepatic and common duct much compressed; cystic duct dilated; a few cancer-spots in liver; other organs healthy. All the cellular tissues were extremely yellow. The lower lobes of the lungs were voluminous and engorged; the seat of the hemorrhage could not be detected. |

| Case | By whom and where reported. | Age and Sex. | Duration. | Local Signs. | Pain. | Vomiting. | State of Bowels. | Jaundice. | Emaciation and Debility. | Dropsy. | Dyspeptic Symptoms. | Other Symptoms, and Remarks. | Post-mortem Appearances. |
|---|---|---|---|---|---|---|---|---|---|---|---|---|---|
| 34 | Dr. Agnew. Proc. of Path. Society, p. 84, or North Am. Med.-Chirg. Rev., July, 1858. | 56; male. | One year; previously in good health. | None. | Pains of flying character, passing through abdomen to the right shoulder; no pain on pressure; feeling of fullness and weight in epigastrium. | Present; matter rejected was of a glairy character, and very offensive. Nausea was among the earliest symptoms, but vomiting occurred only as the case progressed. | Very torpid; evacuations clay-colored; sometimes greenish. | Very slight, if any; (countenance pale and bloodless; color of skin indicating a cancerous cachexia.) | Both markedly present; weight, when first attacked, 250 lbs.; at time of death, 120 lbs. | None. | Signs of indigestion were the first marked symptoms; nausea; flatulency; a feeling of fullness over the stomach. | Troublesome hiccough; he was a good liver, and had, for a long time, had slight dyspeptic symptoms, which became, rather suddenly, much aggravated; at the time they became so he was much depressed in spirits. | Pancreas enlarged to four times its size; its structure replaced by cancerous masses; also a deposit of cancer at cardiac orifice of stomach, (both examined with the microscope;) liver cirrhosed; the ducts pervious; kidneys also seemed granular. The enlarged mass of the pancreas pressed on the thoracic duct. |
| 35 | Dr. Bennett. "Clinical Lectures," p. 449. | 50; male. | One year; previously in good health. | Tumor in epigastrium perceived by patient himself three months and a half before death; tumor very painful on pressure; could be moved upwards and to the right. It was distinctly felt, two inches below the ensiform cartilage, and three above umbilicus. | Pain severe and constant; is epigastric, but not increased on taking food. | Present; occurred at first occasionally; later, became constant; he vomited matter resembling coffee-grounds, mostly one hour to an hour and a half after meals. | Not mentioned. | None. | Both present and progressive. | None. | No appetite; pyrosis as a very early symptom; food could not be retained on stomach; thirst only occasionally; tongue and gums dry. | Tenderness over liver; pulse small and weak; slept but little; urine normal; prolonged expiration, feeble and harsh respiratory murmur in lungs. | Pancreas.—Its head involved with the surrounding mesenteric glands, and a mass compressing the pyloric extremity of the stomach in a cancerous tumor. This mass was seated in the smaller curvature, and projected into the stomach. The remaining portions of the pancreas were healthy, but the duct was obliterated. A cyst in the right kidney; liver felt hard and nodulated; lung presented gelatinous-looking masses, which, microscopically examined, proved to be cancer. |

| | | | | | | | | | | | | | |
|---|---|---|---|---|---|---|---|---|---|---|---|---|---|
| 36 | Dr. Bennett. "Clinical Lectures," p. 462. | 50; male. | Eleven weeks. | None. | Gnawing pain in epigastrium, (was the first symptom of disease;) also acute grinding pain in the region of the liver. | No vomiting; food excited nausea. | Constipated stools of a lead color; at times dark-green. | Yellow tinge of skin appeared four weeks after first symptoms; jaundice steadily increased; finally skin became of a dark, green tint. | Emaciation not mentioned, but progressive exhaustion. | Not mentioned. | Loss of appetite; tongue slightly furred, moist, but became dry; considerable thirst; food excited nausea. | Two weeks after he felt gnawing pain, was overworked; drowsiness, loss of appetite, anorexia appeared, soon followed by jaundice. | Pancreas—right extremity converted into a cancerous tumor, rest of the organ indurated; contained a few small cysts. The portion of the common duct which passed through the tumor barely admitted a small probe. Behind this constriction the common, cystic, and hepatic ducts, were greatly enlarged; liver of a green color; its bile-ducts dilated; some cancerous spots in liver, as also in kidney; gall-bladder distended, containing two gall-stones, supposed, by their passage from the liver, to have occasioned the grinding pain over the organ. |
| 37 | Case communicated to me by Dr. Harris. | 44; colored woman. | Upwards of thirteen months. | None. | Constant, dull, and extending over abdomen. | Of watery fluid, depositing a blackish sediment; never of food; was not as constant as disease advanced; indeed, at one time ceased for several months. | Regular. | Yellowish conjunctiva, but not early in disease, and never very marked. | Both present and progressive. | None. | Present from the first, increased with the disease; loss of appetite marked; tongue remained clean throughout; some flatulency. | Died of exhaustion. | Pancreas converted into a cancerous mass the size of a fist, and having the general characters of encephaloid. Its right extremity was mainly diseased; the liver was slightly enlarged, had a few small, cancerous tumors on its external surface. The stomach was perfectly healthy, so were also the other abdominal organs; common duct, pervious. |

## Figures and Tables

**Fig. 6. f1:**